# Primary Neuroendocrine Tumors of the Endometrium: Management and Outcomes

**DOI:** 10.3389/fonc.2022.921615

**Published:** 2022-06-23

**Authors:** Jingjing Zhang, Li Pang

**Affiliations:** ^1^ Department of Nursing, Shengjing Hospital of China Medical University, Shenyang, China; ^2^ Department of Obstetrics and Gynecology, Shengjing Hospital of China Medical University, Shenyang, China

**Keywords:** SEER, prognostic factors, overall survival, cancer-specific survival, neuroendocrine tumors of the endometrium

## Abstract

**Objective:**

To analyze clinical behavior of, optimal treatment regimens for, outcomes, and prognosis of 170 patients with neuroendocrine tumors (NETs) of the endometrium.

**Methods:**

The Surveillance, Epidemiology, and End Results database was used to identify patients with endometrial NETs diagnosed between 2004 and 2015. Clinical features and treatment regimens were analyzed, and 5-year overall survival (OS) and cancer-specific survival (CSS) were compared among different stages and treatment regimens. Univariate and multivariate analyses were performed to identify independent prognostic factors associated with endometrial NETs. Finally, prognosis was compared between small- and large-cell neuroendocrine carcinoma (SCNEC and LCNEC, respectively) of the endometrium.

**Results:**

There were 20, 8, 47, and 95 patients with stage I, II, III, and IV NET, respectively. The 5-year OS rates of patients in each stage were 59.86%, 42.86%, 32.75%, and 6.04%, respectively. The 5-year CSS survival rates were 59.86%, 50.0%, 38.33%, and 6.39%, respectively. In the multivariate analysis, American Joint Committee on Cancer (AJCC) stage and treatment were associated with poor OS, while AJCC stage, nodal metastasis, and treatment were associated with poor CSS. Neither pathological type nor distant metastasis was associated with prognosis. The rate of distant metastasis was significantly higher for LCNEC than for SCNEC, while 5-year OS and CSS rates were significantly lower.

**Conclusion:**

Complete surgical treatment should be selected regardless of staging for patients with endometrial NETs. For early-stage disease, individualized postoperative treatment with single chemotherapy or radiotherapy may improve OS and CSS. For advanced-stage disease, comprehensive postoperative adjuvant therapy may improve OS and CSS.

## Introduction

Neuroendocrine tumors (NETs) are malignant tumors with neuroendocrine function. NETs occur mainly in the lungs, although they are occasionally observed in the gastrointestinal and genitourinary tracts. Cases of tumors involving the female reproductive tract are rare, with primary NETs of the endometrium accounting for less than 1% of all endometrial cancers (1). In addition to the characteristic histological and immunohistochemical features of NETs, hematogenous and lymphatic metastasis may occur early during the disease in patients with endometrial NETs (2). Furthermore, several studies have reported that endometrial NETs are usually identified in the advanced stage and have a poor prognosis (3–6).

In 2014, the World Health Organization (WHO) classified endometrial NETs as either low-grade or high-grade (3). Low-grade NETs are rarely reported in existing literature (7–9). Nonetheless, low-grade endometrial NETs can be further categorized as either carcinoid or atypical carcinoid (ACT), while high-grade endometrial NETs can be categorized as either small- or large-cell neuroendocrine carcinoma (SCNEC or LCNEC, respectively).

The National Comprehensive Cancer Network (NCCN) has published guidelines concerning treatment strategies for cervical neuroendocrine cancer (10). However, owing to the rarity of endometrial NETs, relevant clinical data from large samples are limited, and standardized treatment options need to be established. To aid in the development of standardized treatment guidelines, the present study aimed to clarify the clinical characteristics, prognosis/prognostic indicators, and outcomes of endometrial NETs.

## Materials and Methods

### Data Collection

Patients histologically diagnosed with NETs of the endometrium from 2004 to 2015 were identified using the SEER database (http://www.seer.cancer.gov; SEER*Stat database: Version 8.3.8) based on the following codes for primary malignant tumors in the endometrium (ICD-O-3/WHO 2008): small-cell carcinoma (8041/3), non-small-cell carcinoma (8046/3), large-cell carcinoma (8012/3), LCNEC (8013/3), atypical carcinoid (8249/3), and carcinoid (8240/3). The exclusion criteria included diagnosis of carcinoma in situ, unknown treatment, unknown survival time, non-endometrial NETs not being the first tumor. Cases were screened for patient-related information, including and clinical characteristics and treatment modality (surgery, chemotherapy, and radiotherapy). Staging was determined in accordance with the American Joint Committee on Cancer (AJCC) staging system. The SEER database is publicly available and contains de-identified data; thus, there was no need to obtain local ethics committee approval for data access.

### Clinical Characteristics

Demographic data including age at diagnosis (<60 years, ≥60 years), year at diagnosis (2004–2009, 2010–2015), AJCC stage (I, IA, IB, IC, INOS; II, IIA, IIB, IINOS; III, IIIA, IIIB, IIIC, IIINOS; IV, IVA, IVB), grade (well/moderately differentiated, poorly/undifferentiated differentiated), lymph node metastasis (negative, positive, not examined, and unknown), sampled pelvic nodes (1–9,10–19, ≥20, not examined, unknown), distant metastases (lung, brain, bone, liver, no, unknown), treatment (surgery alone, chemotherapy [CT] + surgery, radiotherapy [RT] + surgery; concurrent chemoradiotherapy [CCRT] + surgery, CT only, CCRT only; RT only), and surgical approach (curettage, subtotal hysterectomy + adnexectomy, total hysterectomy + adnexectomy + lymphadenectomy, extended radical hysterectomy + adnexectomy + lymphadenectomy, extended radical hysterectomy + adnexectomy + lymphadenectomy + rectal resection, none) were extracted. Data on duration of post-diagnosis follow-up, living status, and cause of death were also extracted from the database to assess OS and CSS, which represented the primary endpoints of the study. For the analysis of OS, death from any cause was considered an event. In the CSS analysis, among the cancer-related deaths, only deaths due to endometrial NETs were considered events. Survival and death from other causes were considered as alive.

### Statistical Analysis

Categorical data are expressed as numbers and percentages (N, %). Pearson’s chi-square analysis was used to analyze the clinical and demographic characteristics of patients with NETs of the endometrium. Kaplan–Meier curves were used to estimate OS and CSS in different groups, and log-rank tests were used to analyze the differences between curves. Univariate and multivariate Cox regression models were used to estimate hazard ratios (HRs) and 95% confidence intervals (CI) for determining the independent prognostic factors associated with OS and CSS. Statistical analysis was performed using SPSS version 25.0 (IBM Corp, Armonk, NY, USA). Kaplan–Meier survival curves were drawn using GraphPad Prism (9.2.0 GraphPad Software, San Diego, CA, USA). P-values < 0.05 were considered statistically significant.

## Results

### Patient Characteristics and Treatment

A total of 170 patients with NETs of the endometrium in the SEER registry met our inclusion criteria, including 56 (32.9%) patients with SCNEC, 60 (35.3%) patients with LCNEC, 2 (1.2%) patients with carcinoid NETs, 1 (0.6%) patient with atypical carcinoid (ACT) NEC, and 51 (30.0%) patients with NETs not otherwise classified. AJCC stage I, II, III, and IV disease was observed in 20 (11.8%), 8 (4.7%), 47 (27.6%), and 95 (55.9%) patients, respectively. **Table 1** presents a more detailed summary of patient characteristics.

**Table 1:** Patient characteristicso of neuroendocrine tumors (NETs)of the endometrium.

Subject	N=170	N(%)
**Hystological type**		
SCNEC	56	32.9
LCNEC	60	35.3
Carcinoid	2	1.2
Atypical carcinoid	1	0.6
NEC(not elsewhere classified)	51	30
**Age(y)**		
<60	52	30.5
≥60	118	69.5
**Year at diagnosis**		
2004-2009	58	34.1
2010-2015	112	65.8
**AJCC stage**		
**I**	**20**	
IA	7	4.1
IB	5	2.9
IC	4	2.4
INOS	4	2.4
**II**	**8**	
**IIA**	2	1.2
IIB	4	2.3
IINOS	2	1.2
**III**	**47**	
IIIA	9	5.3
IIIB	5	2.9
IIIC	32	18.8
IIINOS	1	0.6
**IV**	**95**	
IVA	4	2.3
IVB	91	53.6
**Grade**		
Well/Moderately differentiated	1	0.6
Poorly/undifferentiateddifferentiated	128	75.3
Unknown	41	24.1
**Lymph nodal metastasis**		
Negative	29	17.1
Positive	33	19.4
Not examined	105	61.8
Unknown	3	1.7
**Sampled pelvic nodes**		
1–9	24	14.1
10–19	19	11.2
≥20	20	11.8
Not examined	105	61.8
Unknown	2	1.1
**Distant metastasis**		
bone	13	7.6
brain	8	4.7
liver	16	9.4
lung	23	13.6
No	68	40
Unknown	42	24.7
**Treatment**		
Surgery alone	24	14.1
Surgery + CT	43	25.2
Surgery + CCRT	19	11.1
Surgery + RT	3	1.8
CT alone	25	14.7
CCRT	19	11.2
RT alone	5	3.0
No treatment	32	18.9
**Surgical approach**		
Curettage	1	0.5
Subtotal hysterectomy +Ad	1	0.5
Total hysterectomy+Ad+LN	69	40.6
Extended radical hysterectomy+Ad+LN	18	10.7
No Surgical	81	47.7

RT, radiation; CT, chemotherapy; CCRT, concurrent chemoradiation; N, Number (%); y, years; AJCC, American Joint Commission on Cancer; NOS, not otherwise specified; SCNEC, small cell neuroendocrine carcinoma; LCNEC, large cell neuroendocrine carcinoma; Ad, adnexectomy; LN, lymph node resection.

Bold means p < 0.05.

**Table 2** summarizes the treatments used for each stage of endometrial NETs. Among patients with stage I, II, III, and IV disease, surgery was the main treatment in 15(8.8%), 6(3.5%), 37(21.8%), and 31(18.2%) cases, respectively. Other main treatments included RT only (n=2; beam radiation therapy [EBRT] in 1 case and EBRT with implants in 1 case),combination of EBRT with implants+CT(n=1) for stage I; CT + RT for stage II (n=2;EBRT in 1 case and EBRT with implants in 1 case); CT only (n=2)and EBRT + CT (n=5) for stage III, EBRT only (n=3), EBRT + CT (n=11) and CT only (n=23) for stage IV. Among surgically treated patients with stage I, II, III, and IV disease, treatments included CT in 3, 1, 21, and 18 cases and EBRT + CT in 4, 2, 6, and 4 cases and EBRT with implants in 1, 0, 2, and 0 cases, respectively. Additionally, 1 and 2 patients who underwent surgery for stage I and IV disease received EBRT.

**Table 2:** Treatment at each stage for neuroendocrine tumors (NETs) of the endometrium.

	StageI	StageII	StageIII	StageIV
	n=20	n=8	n=47	n=95
Surgery alone	6	3	8	7
Surgery+CT	3	1	21	18
Surgery+EBRT+CT	4	2	6	4
surgery+combination of EBRT with implants+CT	1	0	2	0
surgery+EBRT	1	0	0	2
EBRT+CT	0	1	5	11
Combination of EBRT with implants+CT	1	1	0	0
Combination of EBRT with implants	1	0	0	0
Implants radiation	0	0	0	0
EBRT	1	0	0	3
CT	0	0	2	23
Not treatment	2	0	3	27

CT, chemotherapy; EBRT, external beam radiation therapy.

### Survival Outcomes

**Figure 1:**
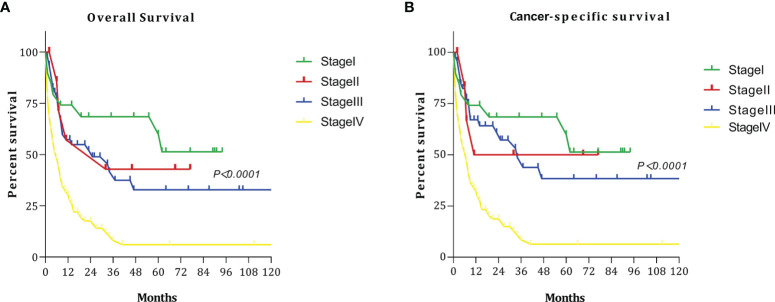
Survival curves at each stage: **(A)** overall survival (OS); **(B)** cancer-specific survival (CSS).

We discussed the survival results of patients with different stages. The OS and CCS curves of patients with different stages are shown in **Figure 1**. The 5-year OS rates for patients with stage I, II, III, and IV disease were 59.86%, 42.86%, 32.75%, and 6.04%, respectively. When stage I was used as the reference, HR for death at stage II, III, and IV were 1.370 (95% CI: 0.3815–4.919), 1.714 (95% CI: 0.845–3.48), and 3.174 (95% CI: 1.875–5.37), respectively. The 5-year CSS rates among patients with stage I, II, III, and IV disease were 59.86%, 50.0%, 38.33%, and 6.39%, respectively. When stage I was used as the reference, the HRs for death at stage II, III, and IV were 1.193 (95% CI: 0.298–4.769), 1.422 (95% CI: 0.663–3.047), and 3.819 (95% CI: 2.335–6.245), respectively.

**Figure 2:**
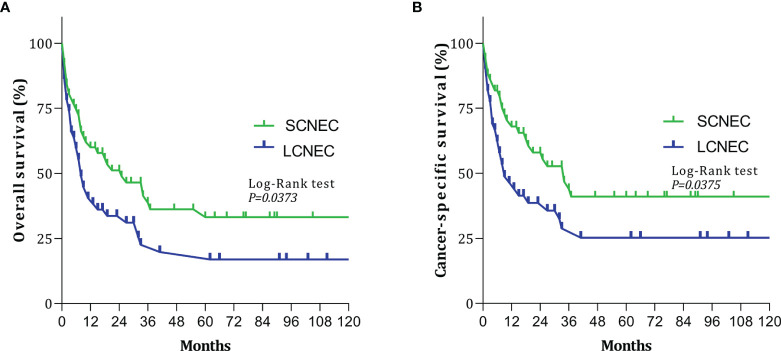
Survival curves for patients with small-cell neuroendocrine carcinoma (SCNEC) and large-cell neuroendocrine carcinoma (LCNEC): **(A)** overall survival (OS); **(B)** cancer-specific survival (CSS).

Since only 1 ACT case and 2 carcinoid cases were identified, comparisons between histological subtypes were restricted to SCNEC and LCNEC. **Figure 2** shows the OS and CSS curves of patients with SCNEC and LCNEC. The median OS time among patients with SCNEC was 25 months, while that among patients with LCNEC was only 8 months. The 5-year OS rates for SCNEC and LCNEC were 33.16% and 16.94%, respectively. Relative to SCNEC, the HR for LCNEC was 1.623 (95% CI: 1.008–2.614, P=0.0373). The 5-year CSS rates for SCNEC and LCNEC were 41.02% and 25.22%, respectively. Relative to SCNEC, the HR for LCNEC was 1.708 (95% CI: 1.011–2.887; P=0.0375). The 5-year OS and CSS rates were thus significantly lower for LCNEC than for SCNEC.

**Figure 3:**
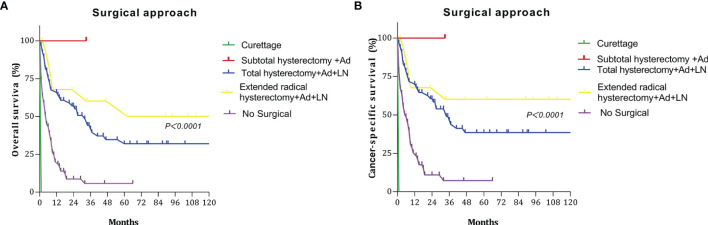
Survival curves at different surgery type: **(A)** overall survival (OS); **(B)** cancer-specific survival (CSS).

We discussed the survival outcomes of patients who underwent different surgeries. The OS and CCS curves of the patients according to surgery type are shown in **Figure 3**. The 5-year OS rates for patients who underwent curettage, subtotal hysterectomy + adnexectomy, total hysterectomy + adnexectomy + lymphadenectomy, extended radical hysterectomy + adnexectomy + lymphadenectomy, and no surgery were 0%, 100%, 32.02%, 50.15%, and 5.80%, respectively. The 5-year CSS rates were 0%, 100.0%, 38.52%, 60.19%, and 7.32%, respectively.

### Prognostic Factors for OS and CSS

To identify factors influencing prognosis among patients with NETs of the endometrium, we selected age, year at diagnosis, AJCC stage, number of lymph nodes sampled, lymph node metastasis, distant metastasis, histological type, and treatment as variables for the univariate and multivariate analyses (**Table 3**). The multivariate analysis showed that AJCC stage and treatment were independent predictors of OS. When stage I was used as the reference, the HR for death in stage III and IV were 3.368 (95% CI: 0.956–11.860) (p=0.039) and 6.750 (95% CI: 1.872–24.345, p=0.004), respectively. When surgery only was used as the reference, the HR for death among the patients who underwent surgery + CT and surgery + CCRT were 0.280 (95% CI: 0.142–0.553) (p <0.001), 0.157 (95% CI: 0.056–0.440) (p <0.001). Meanwhile, AJCC stage, lymph node metastasis, and treatment were independent predictors of CSS. When stage I was used as the reference, the HR for death in stage III and IV were 11.500 (95% CI: 1.259–25.069, p=0.030) and 35.096 (95% CI: 3.673–55.307, p=0.002), respectively. When the lymph node-negative patients were used as the reference, the HR for death in the non-examined lymph node-positive patients were 4.722 (95% CI: 1.552–14.369, p=0.006) and 3.632 (95% CI: 1.027–12.845, p=0.045), respectively. When surgery only was used as the reference, the HR for death in the surgery + CT and surgery + CCRT groups were 0.269 (95% CI: 0.127–0.570, p=0.001) and 0.154 (95% CI: 0.049–0.448, p=0.001), respectively.

**Table 3:** Prognostic factors for neuroendocrine tumors (NETs) of the endometrium.

Subject		Overall survival				Cancer-specific survival			
characteristics		Univariate		Multivariate		Univariate		Multivariate	
	n	HR (95%CI)	p value	HR (95%CI)	p value	HR (95%CI)	p value	HR (95%CI)	p value
**Age**									
<60	52	Ref	0.071	–	–	Ref	0.201	–	–
≥60	118	1.451 (0.968-2.176)				1.318 (0.863-2.013)			
**Year at diagnosis**									
2004-2009	58	Ref	0.094	–	–	Ref	0.149	–	–
2010-2015	112	1.389 (0.946-2.04)		–	–	1.351 (0.898-2.031)		–	–
**AJCC stage**									
I	20	Ref	<**0.001**	Ref	**0.008**	Ref	<**0.001**	Ref	**0.001**
II	8	1.396 (0.420-4.640)	0.586	2.18 (0.457-10.395)	0.328	2.100 (0.470-9.389)	0.332	9.064 (0.769-16.813)	0.080
III	47	1.745 (0.787-3.871)	0.17	3.368 (0.956-11.860)	0.039	2.808 (0.961-8.208)	0.059	11.500 (1.259-25.069)	0.030
IV	95	5.030 (2.400-10.542)	<0.001	6.750 (1.872-24.345)	0.004	9.482 (3.431-26.204)	<0.001	35.096 (3.673-55.307)	0.002
**Sampled pelvic nodes**									
Negative	29	Ref	<**0.001**	Ref	0.099	Ref	**<0.001**	Ref	**0.024**
Positive	33	2.778 (1.264-6.102)	0.011	2.803 (1.078-7.289)	0.035	4.623 (1.728-12.369)	**0.002**	4.722 (1.552-14.369)	0.006
Not examined	105	5.918 (2.945-11.893)	<0.001	1.941 (0.675-5.580)	0.218	9.249 (3.714-23.037)	<0.001	3.632 (1.027-12.845)	0.045
**Lymph node sampling**									
1–9	24	Ref	<**0.001**	Ref	0.594	Ref	<**0.001**	Ref	0.166
10–19	19	0.588 (0.228-1.515)	0.271	1.030 (0.341-3.113)	0.958	0.811 (0.300-2.194)	0.680	2.640 (0.880-7.920)	0.083
≥20	20	0.594 (0.252-1.401)	0.234	1.017 (0.402-2.577)	0.971	0.720 (0.279-1.857)	0.496	1.797 (0.643-5.020)	0.263
Not examined	105	2.378 (1.366-4.138)	0.002	1.921 (0.672-5.582)	0.212	2.866 (1.515-5.421)	0.001	0.769 (0.331-1.790)	0.543
**Distant metastasis**									
Yes	41	Ref	**0.001**	Ref	0.345	Ref	**<0.001**	Ref	0.485
No	68	0.480 (0.305-0.756)	0.002	0.891 (0.476-1.670)	0.720	0.383 (0.236-0.623)	<0.001	0.785 (0.399-1.542)	0.482
Unknown	61	0.469 (0.297-0.742)	0.001	0.645 (0.339-1.228)	0.182	0.434 (0.270-0.696)	0.001	0.670 (0.349-1.287)	0.229
**Hystological type**									
SCNEC	56	Ref	0.055	–	–	Ref	0.059	–	–
LCNEC	60	1.544 (0.991-2.405)		–	–	1.669 (1.026-2.716)		–	–
**Treatment**									
Surgery alone	24	Ref	**<0.001**	Ref	**<0.001**	Ref	**<0.001**	Ref	**0.001**
Surgery + CT	43	0.585 (0.312-1.097)	0.095	0.280 (0.142-0.553)	<0.001	0.643 (0.327-1.266)	0.202	0.269 (0.127-0.570)	0.001
Surgery + CCRT	19	0.206 (0.076-0.560)	0.002	0.157 (0.056-0.440)	<0.001	0.202 (0.066-0.614)	0.005	0.154 (0.049-0.448)	0.001
Surgery + RT	3	0.985 (0.227-4.280)	0.984	1.496 (0.309-7.251)	0.617	1.244 (0.282-5.491)	0.773	2.219 (0.429-11.489)	0.342
CT alone	25	2.066 (1.094-3.904)	0.025	0.664 (0.303-1.457)	0.307	2.371 (1.201-4.679)	0.013	0.627 (0.271-1.451)	0.276
CCRT	19	1.399 (0.691-2.832)	0.351	0.673 (0.300-1.513)	0.339	1.441 (0.671-3.095)	0.349	0.642 (0.267-1.546)	0.323
RT alone	5	2.293 (0.834-6.301)	0.108	1.608 (0.525-4.921)	0.406	2.157 (0.701-6.635)	0.18	1.326 (0.384-4.574)	0.655

AJCC, American Joint Commission on Cancer; SCNEC, small cell neuroendocrine; LCNEC, large cell neuroendocrine carcinoma; HR, hazard ratio; CI, confidence interval.

Bold means p < 0.05.

### Treatment

The main treatment for NETs of the endometrium was surgery, and the most common procedure was hysterectomy + bilateral adnexectomy + pelvic lymphadenectomy in 69 (40.6%) patients, followed by radical total hysterectomy + bilateral adnexectomy + pelvic lymphadenectomy in 18 (10.7%) patients, subtotal hysterectomy + bilateral adnexectomy in 1 (0.5%) patient, and curettage only in 1 (0.5%) patients (**Table 1**). Adjuvant therapy included CT and RT. RT included EBRT, radioactive implants, and EBRT with implants. The SEER database does not provide comprehensive information on CT; it only specifies whether CT was performed, without any specific information. Therefore, in this study, we were unable to determine the CT regimens used or the number of treatments performed.

**Figure 4:**
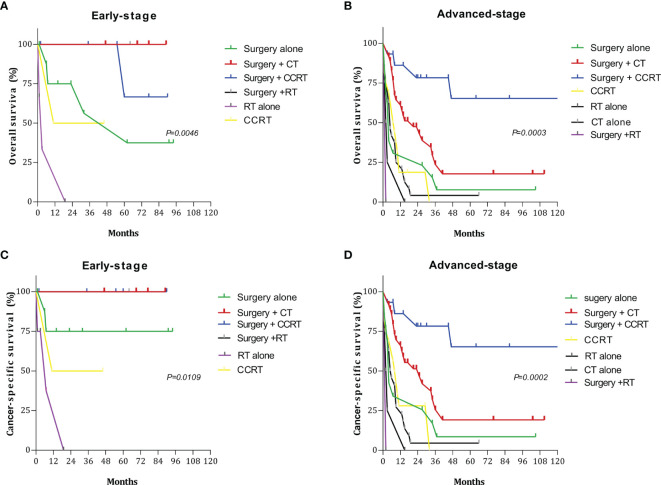
Survival curves for patients with early- and advanced-stage disease for different treatment regimens: **(A)** overall survival (OS) in the early stage; **(B)** overall survival (OS) in the advanced stage; **(C)** cancer-specific survival (CSS) in the early stage; **(D)** cancer-specific survival (CSS) in the advanced stage.

Among the 28 patients with early-stage disease (I and II), the 5-year OS and CSS rates for surgery + CT and surgery + RT were both 100%, which were significantly better than those for other treatment regimens. Among the 142 patients with advanced-stage disease (III and IV), the 5-year OS and CSS rates for surgery + CCRT were both 65.27%. Thus, the survival rates were significantly higher with these treatments than with other treatments (**Figure 4**). The 5-year OS rates and CSS rates for CT only, RT only, and CCRT only were 4.16%, 0%, and 0% and 4.55%, 0%, and 0%, respectively (**Table 4**).

**Table 4:** Five-year OS and CSS according to stage and treatment in patients with neuroendocrine tumors (NETs) of the endometrium.

Treatments	N	5-year OS	*P*	5-year CSS	P
**Stage I-II**			0.0046		0.0109
Surgery alone	9	37.50%		75.00%	
Surgery + CT	4	100.00%		100.00%	
Surgery + CCRT	6	66.67%		100.00%	
Surgery + RT	1	100.00%		100.00%	
CCRT	3	50.00%		50.00%	
RT alone	2	0.00%		0.00%	
CT alone	0	0		0	
**Stage III-IV**			0.0003		0.0002
Surgery alone	15	7.69%		8.54%	
Surgery + CT	39	17.76%		19.23%	
Surgery + CCRT	13	65.27%		65.27%	
Surgery + RT	2	0.00%		0.00%	
CCRT	16	0.00%		0.00%	
RT alone	3	0.00%		0.00%	
CT alone	25	4.16%		4.55%	

RT, radiotherapy; CT, chemotherapy; CCRT, concurrent chemoradiotherapy; OS, overall survival; CSS, cancer-specific survival.

## Discussion

Endometrial NETs is a rare disease with poor prognosis. Given its extremely low incidence, the most effective methods for treating endometrial NETs and the most important factors for determining prognosis remain unknown, making clinical management difficult. In addition, due to its rarity, there are no evidence-based standards or international guidelines for the diagnosis and treatment of endometrial NETs. Therefore, we utilized the large sample size of the SEER database to investigate clinical features, prognosis, and treatment options for NETs of the endometrium.

Because NETs of the endometrium are very rare, the existing reports include case reports and small case series (4, 5, 7–16). The largest previous study analyzed data for 42 cases of endometrial NETs occurring in Japan over a 19-year period (17). This multicenter study suggested that stage III–IV disease and pure SCNEC are associated with significantly poorer prognosis than other disease stages and histological types. However, some studies have reported long-term survival in patients with advanced disease (10, 12, 16). Sawada et al. (17) reported a rare case of advanced SCNEC with liver and brain metastases in a patient who underwent pelvic tumor reduction surgery + metastatic resection and postoperative treatment with CT (irinotecan + cisplatin) + RT, following which the patient survived for 12 years. Viau et al. (18) reported a case of stage IV SCNEC treated with surgery + CT (cisplatin + etoposide) + RT, and their patient remained alive 5 years later.

The current study included the largest cohort of patients with NETs of the endometrium to date. Given its large sample size relative to previous reports (170 cases), our study provides stronger evidence that surgery should be the main treatment strategy regardless of the endometrial NET stage. In addition, our results suggest that for early-stage disease, individualized postoperative treatment *via* single CT or radiotherapy may improve OS and CSS. For advanced-stage disease, comprehensive postoperative adjuvant therapy may improve OS and CSS. Since only one patient underwent subtotal hysterectomy + adnexectomy, it is necessary to continue accumulating cases for further analyses. From our analysis, the 5-year OS and CSS of patients who underwent total hysterectomy + adnexectomy + lymphadenectomy and extended radical hysterectomy + adnexectomy + lymphadenectomy are higher than those of patients who underwent other treatment methods. Therefore, complete surgical treatment may improve outcomes in patients with the disease.

Nonetheless, comprehensive treatment may not enable long-term survival in all patients with NETs of the endometrium, especially those with LCNEC. Tu (19) reported a case of stage IVB LCNEC treated with adjuvant CT (cisplatin + etoposide) postoperatively, following which a cisplatin + ifosfamide regimen was used to treat disease progression. Two months later, obstructive ileus was observed, and the patient underwent second surgery. However, she died of infection 8 days after surgery. Kobayashi (20) reported a case of stage IIIC2 LCNEC in which CCRT was initiated 1 month after surgery. The patient developed rapidly progressing metastases in the upper abdominal and cervical regions subsequently and died eventually of the disease 309 days after surgery.

Based on the treatment plan for pulmonary NETs, platinum-based CT is often used for adjuvant treatment in patients with NETs of the endometrium. Currently, the most common regimen is paclitaxel + carboplatin, followed by EP (cisplatin + etoposide) and other treatment options. EBRT, implants, or a combination of EBRT and implants is recommended for RT. Some researchers have suggested that CT is also required in the early stage given the aggressive nature of NETs of the endometrium (4, 21). Korcum et al. (22) argued that brachytherapy may be sufficient when performed in conjunction with cisplatin treatment to prevent systemic micrometastases. NETs of the endometrium often presents with disseminated disease, indicating that radical surgery with CT would be appropriate for both early and advanced cases (1). Combined treatment with CT and somatostatin-like octreotide has also been reported in patients with NETs of the endometrium. The inhibitory effect of somatostatin analogs on tumor growth has been demonstrated (23).

To date, no studies have characterized the specific imaging findings associated with endometrial NETs. Makihara et al. (24) reported that MRI findings for LCNEC were similar to those for other poorly differentiated endometrial carcinomas and sarcomas, and preoperative diagnosis of endometrial NETs based on MRI or PET/CT remains difficult (25).

Previous studies analyzing the relationship between prognosis and histological subtypes of endometrial NETs have yielded contradictory conclusions. In this study, we compared the prognoses of SCNEC and LCNEC. Several studies have indicated that SCNEC is the most common histological subtype of endometrial NETs (1, 5, 6, 16, 26–28). While some authors have reported worse prognosis for SCNEC than for LCNEC (8), others have reported that LCNEC tends to be more aggressive and has a worse prognosis than SCNEC (1, 6, 29). Furthermore, Mulvany et al. (27) reported very poor prognosis among patients with LCNEC regardless of stage. These discrepancies are likely due to the small sample size. In this study, we compared data for 56 cases of SCNEC and 60 cases of LCNEC. The median survival time for SCNEC was 25 months, while that for LCNEC was only 8 months. The prognosis of LCNEC is significantly lower than that of SCNEC. These findings may help to clarify the influence of histological subtype on prognosis in patients with endometrial NETs.

Common metastasis sites in patients with NETs of the endometrium include the brain, lungs, liver, kidney, and bone; and NETs of the endometrium often has rapid metastasis and recurrence (27, 30, 31). Our study found that distant metastasis sites of NETs of the endometrium were the brain, lungs, liver, and bone, accounting for 35.3% of all cases, and there was no information regarding recurrence in the SEER database. To improve the prognosis of recurrent endometrial NETs, future studies focusing on early detection techniques and optimal strategies for managing recurrence are warranted.

This article has certain limitations. First, while the SEER database informs whether patients received CT, it does not specify the type of CT or the number of CT/RT cycles, highlighting the need for further studies to determine which regimens are most effective at each disease stage. The SEER database has other limitations, as it does not provide details related to the time of treatment, the treatment location, or the treatments used in cases of recurrence. Additional clinical cases must be accumulated to address these issues. Moreover, there are currently no standard treatment options for recurrent NETs of the endometrium. Although molecular typing focuses on endometrial non-neuroendocrine carcinomas, novel drug treatments based on molecular targeting represent a key area of research. Nonetheless, there is currently no method for molecular typing that can aid in identifying prognostic subgroups among patients with NETs of the endometrium (32), and only one study has demonstrated the role of mismatch repair proteins in endometrial NETs (6).

## Conclusion

Our findings indicate that AJCC stage and treatment are independent prognostic factors for OS, while AJCC stage, nodal metastasis, and treatment are independent prognostic factors for CSS. Complete surgical treatment may improve outcomes in patients with the disease. For patients with early NETs of the endometrium, treatment regimens including surgery and postoperative adjuvant RT or CT can significantly improve OS and CSS. For patients with advanced NETs of the endometrium, surgery should be selected as the primary treatment method when feasible, and postoperative adjuvant comprehensive therapy (surgery + CT + RT) may help to improve OS and CSS. Further studies are required to determine the most appropriate treatment regimens and prognostic factors for recurrent endometrial NETs.

## Data Availability Statement

The datasets presented in this study can be found in online repositories. The names of the repository/repositories and accession number(s) can be found in the article/supplementary material.

## Author Contributions

JZ collected clinical data and wrote the manuscript; Conceptualization; Data curation; Formal analysis; Writing - original draft. LP Data curation; Writing - review and editing. Both authors contributed to the article and approved the submitted version.

## Conflict of Interest

The authors declare that the research was conducted in the absence of any commercial or financial relationships that could be construed as a potential conflict of interest.

## Publisher’s Note

All claims expressed in this article are solely those of the authors and do not necessarily represent those of their affiliated organizations, or those of the publisher, the editors and the reviewers. Any product that may be evaluated in this article, or claim that may be made by its manufacturer, is not guaranteed or endorsed by the publisher.

## Abbreviations

ACT, atypical carcinoid; AJCC, American Joint Committee on Cancer; CCRT, concurrent chemoradiotherapy; CI, confidence interval; CSS, cancer-specific survival; CT, chemotherapy; EBRT, external beam radiation therapy; HR, hazard ratio; LCNEC, large-cell neuroendocrine carcinoma; NETs, neuroendocrine tumors; OS, overall survival; RT, radiotherapy; SEER, Surveillance, Epidemiology, and End Results; SCNEC, small-cell neuroendocrine carcinoma; WHO, World Health Organization.
